# Identifying treatment response to antihypertensives in patients with obesity-related hypertension

**DOI:** 10.1186/s40885-017-0077-x

**Published:** 2017-10-24

**Authors:** Ilse M. Schrover, Jannick A. N. Dorresteijn, Jodine E. Smits, A. H. Jan Danser, Frank L. J. Visseren, Wilko Spiering

**Affiliations:** 1 Department of Vascular Medicine, University Medical Center Utrecht, P.O. Box 85500, 3584 GA Utrecht, The Netherlands; 2 Division of Pharmacology and Vascular Medicine, Department of Internal Medicine, Erasmus Medical Center, Rotterdam, The Netherlands

**Keywords:** Hypertension, Obesity, Treatment, Interaction

## Abstract

**Background:**

In patients with obesity-related hypertension (ORH), reaction to antihypertensive medication is likely influenced by patientcharacteristics.

**Methods:**

Effects of aliskiren, moxonidine and hydrochlorothiazide on 24-h blood pressure (BP) were compared to placebo. Linear mixed effect models were used to analyze the effect of patient characteristics on BP levels and treatment response.

**Results:**

Systolic BP response to aliskiren was higher in patients with a BMI > 30.7 kg/m2 compared to patients with a BMI ≤ 30.7 kg/m2 (−21 mmHg versus -4 mmHg). In patients with a hsCRP > 1.8 mg/L the systolic BP response to aliskiren was higher than in patients with a low hsCRP (−15 mmHg versus −7 mmHg). Hydrochlorothiazide (HCTZ) treatment effect on systolic BP was −13 mmHg when heart rate > 71 beats/min compared to -3 mmHg when heart rate was ≤ 71 beats/min.

**Conclusion:**

In patients with ORH, BP response to aliskiren is positively related to BMI and hsCRP. Systolic BP response to HCTZ is positively related to heart rate and negatively to renin levels.

**Trial registration:**

NCT01138423. Registered June 4th, 2010.

## Background

Subjects who are either overweight or obese have a 2 time higher risk of developing hypertension as compared to persons with a normal BMI [1, 2]. In patients with this so-called obesity-related hypertension (ORH), both blood pressure level as well as the reaction to anti-hypertensive medication are likely to be influenced by patient characteristics. Age and gender are known to influence blood pressure levels in the general population and are likely to also influence blood pressure in patients with ORH. At age 55, 7% of the Western population is diagnosed with hypertension [1], which rises to 34% at age 65 and 77% at age 80 [3]. Women (72%) are more likely to develop hypertension than men (61%) during their lifetime [3].

Patient characteristics linked to the underlying pathophysiological mechanisms of ORH could also influence blood pressure. The major underlying mechanism linking obesity to hypertension is thought to be adipose tissue dysfunction, defined as an imbalance between the release of pro- and anti-inflammatory adipokines, resulting in activation of the renin-angiotensin-aldosterone system (RAAS), sympathetic overdrive, low-grade inflammation and oxidative stress [4–7]. Eventually, this leads to endothelial dysfunction, vascular hypertrophy and impaired natriuresis [4, 8]. In a previous study, we showed that in subjects with ORH the blood pressure lowering effect was greatest during treatment with direct renin inhibitor aliskiren [9].

Knowledge of patient characteristics that influence the blood pressure response to antihypertensive treatment in patients with ORH may help to identify the most effective blood pressure lowering treatment in individual patients. Response to anti-hypertensive medication varies significantly in patients with previously untreated primary hypertension [10–12] and is therefore presumably dependent on patient-specific characteristics such as gender, age, BMI, salt intake and level of RAAS hormones [12–18].

In the present study we evaluated which patient characteristics influence the blood pressure-lowering effect of the direct renin inhibitor aliskiren, the sympathicolytic agent moxonidine and the diuretic hydrochlorothiazide in patients with ORH.

## Methods

We used data from a cross-over trial in which 31 previously untreated patients with ORH were successively treated with aliskiren, moxonidine, hydrochlorothiazide (HCTZ) and matching placebo, in random order, each for 8 weeks. A detailed description of the methods has been published earlier [9]. In summary, a four-way, double-blind, single-center, crossover study was performed in 31 adult Caucasian patients (men and post menopausal women) with previously untreated ORH from September 2010 until March 2012. ORH was defined as a blood pressure > 130 mmHg systolic and/or > 85 mmHg diastolic and abdominal obesity (waist circumference > 102 cm (men) or >88 cm (women)). This is in accordance with the metabolic syndrome criteria, which all patients fulfilled [19].

(Pre)hypertension was defined as office systolic blood pressure (SBP) > 130 mmHg and/or office diastolic blood pressure (DBP) > 85 mmHg during two screening visits on separate days. Blood pressure was measured two times on both arms in sitting position after the subject had been seated for some minutes using an appropriately sized arm-cuff and an automated oscillometric blood pressure device [20]. Blood pressure level was defined as the highest mean of the measurements on one arm during the first visit, and as the mean of the measurements on that same arm during all subsequent visits. When subjects were eligible to participate in the study they entered a 40-weeks study-period in which they received each of four subsequent once daily monotherapies sequentially: aliskiren 300 mg, moxonidine 0.4 mg, HCTZ 25 mg, and matching placebo. The efficacy of each treatment on ambulatory (24-h) blood pressure was assessed after 8 weeks. The study was conducted in accordance with the principles of the Declaration of Helsinki as adopted by the 59th WMA general assembly, Seoul 2008. The institutional review committee of the University Medical Center Utrecht approved the study and all patients gave their written informed consent.

### Data analyses

A linear mixed effect model was used to determine which characteristics modified the relationship between treatment (aliskiren, moxonidine or HCTZ) and 24-h systolic and diastolic blood pressure (DBP), adjusted for time since randomization, age and gender. Patient characteristics considered were gender, BMI, waist, age, fasting glucose, mean 24-h heart rate, aldosterone, renin, muscle sympathetic nerve activity (MSNA), 24-h urine sodium excretion, hsCRP, adiponectin and leptin. These characteristics were measured during placebo treatment (in fasting condition between 7 and 9 am), except BMI and waist circumference, which were only measured at study baseline. MSNA was only performed in a subgroup of 15 patients due to the invasiveness and time-consuming nature of the measurement. Adiponectin, leptin and hsCRP levels were logtransformed due to their skewed distribution. All available follow-up data were included in the analyses even when patients did not complete all four treatments. The 95% confidence intervals (95% CI) were approximated on the basis of the coefficients’ standard errors.

In the analysis determining characteristics influencing reaction to medication, scale variables were dichotomized at the median level and treatment effect was presented accordingly, combined with *p*-values for interaction. Analyses were performed with the statistical package R, version 2.11.1 (R Foundation for Statistical Computing, www.R-project.org).

## Results

### Baseline characteristics

Patients consisted of 23 men and 8 women with a median age of 60 years (IQR 55-63 years), a median BMI of 30.7 kg/m^2^ (IQR 27.7-32.2 kg/m^2^) and a median office blood pressure of 153/88 mmHg (IQR 145-167 mmHg systolic and 84-96 mmHg diastolic; Table 1).

**Table 1:** Patient characteristics during treatment with placebo

Characteristic		*N* = 31
Sex	Men (n, %)	23 (74%)
	Women (n, %)	8 (26%)
Age (years)		60 (55-63)
BMI (kg/m^2^)^a^		30.7 (27.7–32.2)
Waist circumference (cm)^a^	Men	111 (107–116)
	Women	98 (95–99)
Office blood pressure (mmHg)	Systolic	153 (145–167)
	Diastolic	88 (84–96)
24-h blood pressure (mmHg)	Systolic	131 (124–144)
	Diastolic	80 (76–87)
Heart rate (beats/min)		71 (69–79)
MSNA (bursts/min)		46 (39–50)
Sodium intake (mmol/day)		87 (58–118)
Fasting glucose (mmol/L)		5.6 (5.0–6.0)
Total cholesterol (mmol/L)		5.7 (5.1–6.2)
HDL-cholesterol (mmol/L)	Men	1.02 (0.94–1.22)
	Women	1.31 (1.28–1.76)
Triglycerides (mmol/L)		1.90 (1.40–2.30)
hsCRP (mg/L)		1.8 (1.3–3.3)
eGFR (ml/min/1.73m^2^)		79 (73–86)
Renin (pg/ml)		57.2 (42.4–79.4)
Aldosterone (pg/ml)		53.4 (33.2–76.4)
Adiponectin (μg/ml)		68.9 (51.9–104.1)
Leptin (ng/ml)		16.4 (9.5–33.2)

Data are presented as medians and interquartile ranges unless otherwise indicated

*BMI* body mass index, *HDL-cholesterol* high density lipoprotein cholesterol, *eGFR* Glomerular Filtration Rate, as estimated by the Modification of Diet in Renal Disease (MDRD) equation, *MSNA* muscle sympathetic nerve activity, *hsCRP* high sensitivity C reactive protein

^a^BMI and waist circumference were only measured at baseline

### Determinants of blood pressure response to aliskiren

Table 2 shows the relation between patient characteristics and 24-h SBP in response to treatment with the RAAS-inhibitor aliskiren.

**Table 2:** Effect of RAAS inhibition (aliskiren 300 mg) on 24-h SBP

		24-h SBP	P for interaction
Sex	Men	−13 (−18 to −8)	0.75
	Women	−4 (−7 to −2)	
Age	> 60 years	−7 (−12 to −2)	0.88
	≤ 60 years	−11 (−15 to −6)	
BMI	> 30.7 kg/m2	−21 (−27 to −14)	0.01
	≤ 30.7 kg/m2	−4 (−9 to 1)	
Waist (sexpooled)	Men > 111 cm Women >98 cm	−17 (−25 to −9)	0.39
	Men ≤ 111 cm Women ≤ 98 cm	−10 (−14 to −5)	
Heart rate^b^	> 71/min	−14 (−20 to −7)	0.71
	≤ 71/min	−9 (−14 to −4)	
MSNA	> 46 bpm	−9 (−17 to 1)	0.16
	≤ 46 bpm	−9 (−15 to −3)	
hsCRP^c^	> 1.8 mg/L	−15 (−20 to −10)	0.03
	≤ 1.8 mg/L	−7 (−12 to −2)	
Renin	> 57.2 pg/ml	−16 (−22 to −9)	0.73
	≤ 57.2 pg/ml	−9 (−14 to −3)	
Aldosterone	> 53.4 pg/ml	−14 (−21 to −7)	0.75
	≤ 53.4 pg/ml	−11 (−16 to −6)	
Sodium intake^a^	> 87 mmol/day	−14 (−20 to −7)	0.48
	≤ 87 mmol/day	−9 (−14 to −4)	
Glucose	> 5.1 mmol/L	−20 (−26 to −13)	0.13
	≤ 5.1 mmol/L	−8 (−13 to −3)	
Adiponectin^c^	> 68.9 μg/ml	−11 (−16 to −6)	0.56
	≤ 68.9 μg/ml	−13.3 (−20 to −7)	
Leptin^c^	> 16.4 ng/ml	−16 (−21 to −10)	0.81
	≤ 16.4 ng/ml	−8 (−13 to −2)	

BP change (95% confidence interval) in mmHg from placebo in subgroups of patients corrected for age and gender. Patient characteristics were dichotomized on the median level and p’s for interaction were determined accordingly

*SBP* systolic blood pressure, *BMI* body mass index, *MSNA* muscle sympathetic nerve activity, *hsCRP* high sensitivity C-reactive protein, *bpm* beats per minute﻿, *ABPM* ambulatory blood pressure measurement﻿

^a^Estimated on the basis of 24-h sodium excretion in urine

^b^Measured with ABPM

^c^Log transformed

SBP response to aliskiren was significantly related to BMI. SBP decreased with 21 mmHg (95% CI −27 to −14 mmHg) in patients with a BMI above the median of 30.7 kg/m^2^ versus a decrease of 4 mmHg (95% CI −9 to 1 mmHg) in those with a BMI below the median, (*p* for interaction = 0.01).

SBP response was also significantly related to hsCRP in patients with > 1.8 mg/L (−15 mmHg, 95% CI −20 to −10 mmHg) compared with patients with ≤ 1.8 mg/L (−7 mmHg, 95% CI −12 to −2 mmHg, p for interaction = 0.03). None of the other patient characteristics was an effect modifier in the relation between treatment with aliskiren and blood pressure reduction.

### Determinants of blood pressure response to hydrochlorothiazide

Table 3 shows the relation between patients characteristics on 24-h SBP response to diuretic therapy with hydrochlorothiazide. During treatment with HCTZ, blood pressure response was significantly related to resting heart rate. When heart rate was > 71 beats/min there was a decrease of 13 mmHg (95% CI −19 to −7 mmHg) in SBP as opposed to a decrease of 3 mmHg (95% CI −7 to 2 mmHg) in SBP when heart rate was ≤ 71 beats/min (p for interaction = 0.03). Renin level was also an effect modifier in the relation between treatment with HCTZ and blood pressure reduction. Renin levels below the median of 57.2 pg/ml were related to larger SBP reductions (−8 mmHg, 95% −13 to −3 mmHg) than renin levels above the median (−6 mmHg, 95% CI −12 to 1 mmHg; p for interaction = 0.04). None of the other patient characteristics was an effect modifier in the relation between treatment with HCTZ and blood pressure reduction.

**Table 3:** Effect of diuretic therapy (HCTZ 25 mg) on 24-h SBP

		24-h SBP	P for interaction
Sex	Men	−7 (−12 to −2)	0.35
	Women	−4 (−7 to −2)	
Age	> 60 years	−4 (−8 to 1)	0.74
	≤ 60 years	−7 (−12 to −3)	
BMI	> 30.7 kg/m2	−11 (−18 to −5)	0.99
	≤ 30.7 kg/m2	−4 (−9 to 1)	
Waist (sexpooled)	Men > 111 cm Women >98 cm	−8 (−16 to 1)	0.79
	Men ≤ 111 cm Women ≤ 98 cm	−6 (−11 to −1)	
Heart rate^b^	> 71/min	−13 (−19 to −7)	0.03
	≤ 71/min	−3 (−7 to 2)	
MSNA	> 46 bpm	−6 (−14 to 3)	0.17
	≤ 46 bpm	−8 (−14 to −1)	
hs-CRP^c^	> 1.8 mg/L	−8 (−14 to −3)	0.70
	≤ 1.8 mg/L	−5 (−10 to 0)	
Renin	> 57.2 pg/ml	−6 (−12 to 1)	0.04
	≤ 57.2 pg/ml	−8 (−13 to −3)	
Aldosterone	> 53.4 pg/ml	−8 (−15 to −1)	0.54
	≤ 53.4 pg/ml	−7 (−12 to −2)	
Sodium intake^a^	> 87 mmol/day	−7 (−13 to 0)	0.42
	≤ 87 mmol/day	−8 (−12 to −3)	
Glucose	> 5.1 mmol/L	−8 (−15 to −2)	0.74
	≤ 5.1 mmol/L	−6 (−11 to −1)	
Adiponectin^c^	> 68.9 μg/ml	−8 (−13 to −3)	0.94
	≤ 68.9 μg/ml	−6 (−13 to 1)	
Leptin^c^	>16.4 ng/ml	−10 (−16 to −4)	0.29
	≤16.4 ng/ml	−4 (−10 to 1)	

BP change (95% confidence interval) in mmHg from placebo in subgroups of patients corrected for age and gender. Patient characteristics were dichotomized on the median level and p’s for interaction were determined accordingly

*SBP* systolic blood pressure, *BMI* body mass index, *MSNA* muscle sympathetic nerve activity, *hsCRP* high sensitivity C-reactive protein, *bpm* beats per minute, *ABPM* ambulatory blood pressure measurement

^a^Estimated on the basis of 24-h sodium excretion in urine

^b^Measured with ABPM

^c^Log transformed

### Determinants of blood pressure response to moxonidine

Table 4 shows the relation between participant characteristics and change in 24-h SBP in response to sympatholytic therapy with moxonidine.

**Table 4:** Effect of sympatho-inhibition (moxonidine 0.4 mg) on 24-h SBP

		24 h SBP	P for interaction
Sex	Men	−3 (−8 to 3)	0.06
	Women	−4 (−7 to −1)	
Age	> 60 years	−2 (−7 to 3)	0.09
	≤ 60 years	−4 (−9 to 1)	
BMI	> 30.7 kg/m2	−4 (−11 to 3)	0.91
	≤ 30.7 kg/m2	−3 (−8 to 2)	
Waist (sexpooled)	Men > 111 cm Women >98 cm	−2 (−10 to 7)	0.84
	Men ≤ 111 cm Women ≤ 98 cm	−4 (−9 to 1)	
Heart rate^b^	> 71/min	−8 (−15 to - 2)	0.53
	≤ 71/min	−1 (−5 to 4)	
MSNA	> 46 bpm	0 (−9 to 9)	0.15
	≤ 46 bpm	−7 (−14 to 0)	
hs-CRP^c^	> 1.8 mg/L	−5 (−10 to 1)	0.46
	≤ 1.8 mg/L	−2 (−7 to 3)	
Renin	> 57.2 pg/ml	−2 (−9 to 5)	0.07
	≤ 57.2 pg/ml	−5 (−11 to 0)	
Aldosterone	> 53.4 pg/ml	−4 (−12 to 3)	0.84
	≤ 53.4 pg/ml	−4 (−9 to 1)	
Sodium intake^a^	> 87 mmol/day	−4 (−11 to 4)	0.25
	≤ 87 mmol/day	−4 (−9 to 1)	
Glucose	> 5.1 mmol/L	−5 (−12 to 2)	0.81
	≤ 5.1 mmol/L	−3 (−8 to 2)	
Adiponectin^c^	> 68.9 μg/ml	−5 (−10 to 1)	0.31
	≤ 68.9 μg/ml	−4 (−11 to 3)	
Leptin^c^	> 16.4 ng/ml	−6 (−11 to 1)	0.45
	≤ 16.4 ng/ml	−2 (−8 to 4)	

BP change (95% confidence interval) in mmHg from placebo in subgroups of patients corrected for age and gender. Patient characteristics were dichotomized on the median level and p’s for interaction were determined accordingly

*SBP* systolic blood pressure, *BMI* body mass index, *MSNA* muscle sympathetic nerve activity, *hsCRP* high sensitivity C-reactive protein, *bpm* beats per minute, *ABPM* ambulatory blood pressure measurement

^a^Estimated on the basis of 24-h sodium excretion in urine

^b^Measured with ABPM

^c^Log transformed

During moxonidine use, women (−4 mmHg SBP, 95% CI −7 to −1 mmHg) tended to have larger blood pressure reductions than men (−3 mmHg SBP, 95% CI −8 to 3 mmHg; p for interaction = 0.06).

Also, participants ≤ 60 years (−4 mmHg, 95% CI −9 to 1 mmHg) compared with participants > 60 years (−2 mmHg, 95% CI −7 to 3 mmHg; p for interaction = 0.09) tended to have larger reductions in SBP.

None of the other patient characteristics was an effect modifier in the relation between treatment with moxonidine and blood pressure reduction.

### Sensitivity analyses

In patients with a screening blood pressure of > 140/90 mmHg, results were not significantly different from those in the patients with a screening blood pressure > 130/85 mmHg.

## Discussion

In patients with obesity-related hypertension the characteristics BMI and hsCRP influence the blood pressure lowering effect of the RAAS inhibitor aliskiren. Heart rate and renin levels influence the effect of the diuretic HCTZ and age and gender influence the effect of the sympatho-inhibitor moxonidine.

The positive association between adipose tissue and blood pressure is mainly driven by visceral adipose tissue in males and by adipose tissue deposited elsewhere in females [16]. As a reaction to accumulation of visceral adipose tissue there is enhanced sympathoactivation, driven partly by testosterone levels. Therefore, blocking sympathoactivation might be more beneficial in reducing blood pressure in men than in women. Although women have lower sympathoactivation than men at all ages, women show a greater increase in sympathetic activity with increasing age [21, 22], followed by a more marked increase in mean blood pressure, suggesting that sympathetic activity has a greater influence on blood pressure in women than in men [21]. MSNA and, to a lesser extent, heart rate are indicative for sympathetic activation. Unexpectedly, we did see the best reaction to moxonidine on SBP in the younger patients. Although a standard maintenance dose of moxonidine (0.4 mg once daily) was given, this might not have blocked all sympathetic activity, especially in those patients with high baseline sympathetic activity. This might also explain why MSNA did not modify the relationship between moxonidine and 24-h SBP, in combination with the relatively small number of patients [15] undergoing MSNA. Unexpectedly, heart rate was found to influence SBP when patients were treated with HCTZ, which does not exhibit sympatho-inhibitory effects [23]. To our knowledge, this has not been reported previously.

One of the other mechanisms underlying ORH is an inappropriately normal or even elevated RAAS activity. An increasing BMI is correlated to an augmented activity of the RAAS system [24, 25] as reflected in higher levels of angiotensinogen, renin, angiotensin I and angiotensin converting enzyme (ACE) in obese persons as compared to lean persons [24–26]. Independent adipose tissue production of angiotensinogen [27] and factors stimulating the adrenal gland in producing aldosterone [28] are presumably responsible for this enhanced activity in obese individuals. The synthesis and secretion of angiotensinogen in adipose tissue does not only contribute to elevated local angiotensin II concentrations, causing oxidative stress and local inflammation, but also lead to higher systemic RAAS activity [7].

Renin and aldosterone levels drop significantly with weight loss, even when the BMI remains > 25 kg/m^2^, suggesting that there is a linear association between BMI and RAAS-activation. Inhibiting RAAS could therefore be more effective in terms of blood pressure reduction in individuals with the highest BMI [13]. Aliskiren, being a direct renin-inhibitor, is known to reduce RAAS activity at both the systemic level [29] and also penetrates the adipose tissue at levels sufficient to reduce tissue RAAS activity [30]. Since enhanced inflammation is, at least partly, a reflection of locally enhanced RAAS activation [31], this may explain the higher blood pressure reductions in patients with the highest hsCRP plasma concentrations. Unexpectedly, blood pressure lowering response to aliskiren was not influenced by either renin or aldosterone plasma concentrations, nor was there an influence of renin and aldosterone plasma concentrations on blood pressure level, despite the known inhibitory effect of aliskiren on both systemic and adipose tissue RAAS activity [29, 30]. There is limited evidence supporting a role for measuring RAAS hormones as guidance for anti-hypertensive therapy. Small retrospective studies show conflicting results concerning the modifying effects of plasma renin activity (PRA) on the blood pressure lowering effect of RAAS inhibitors [32, 33]. A small prospective study shows PRA to correlate with blood pressure reduction in patients treated with angiotensin receptor blockers [34]. In our study we did not measure PRA but plasma renin concentration (PRC), but both parameters are highly correlated in untreated individuals [35].

In ORH, due to impaired pressure natriuresis, volume overload can be an underlying pathophysiological phenomenon [4]. Both higher age and low renin levels are associated with a high intravascular volume status. Thus, older patients and those with low renin levels have a larger blood pressure reduction in response to diuretic therapy [12, 14, 17].

In this study, patients with the lowest plasma renin concentrations, an indication for high volume status, responded most to HCTZ therapy with respect to systolic blood pressure lowering. This is in line with observations in overweight hypertensive patients [14]. Contrary to our expectations, no influence on blood pressure response of sodium intake in either of the treatment arms was seen. For patients treated with aliskiren, 1 earlier study has shown that those on a low salt diet (< 5 g NaCl/day) experience the greatest antihypertensive effect [18] compared to patients on a normal to high salt diet (> 10 g NaCl/day), with a blood pressure difference of 9.4 mmHg. The same beneficial effect of a low sodium diet has been reported for ACE inhibitors and angiotensin receptor blockers [15]. A less prominent effect of low sodium diet is seen when patients are treated with other classes of antihypertensive drugs (calciumblockers, betablockers and diuretics), approximately 4 mmHg blood pressure difference between a low and high sodium diet [15]. In both studies, a large difference in sodium intake was created, either by using a cross-over design with one period of low and one period of high sodium intake or by using a ‘normal’ sodium intake control group opposed to a group instructed to adhere to a low sodium diet. In our study, no such contrast was created and since patients were asked to adhere to a normal salt diet, a relatively low sodium intake (5.1 g NaCl/day) was observed, which might explain why we did not find sodium intake to influence blood pressure response. Moreover, those studies did not exclusively study patients with ORH, although the mean BMI in both studies was > 25 kg/m2.

Strengths and limitations of our study need to be considered. The influence on blood pressure response of possible effect modifiers could be analyzed during treatment with three different classes of antihypertensive medication, aimed at different pathophysiological mechanisms. Blood pressure and heart rate were measured very accurately with 24-h ambulant blood pressure measurements. An important limitation of this study is the relatively small sample size, especially the amount of women participating in the trial. In this study, participants were regarded hypertensive with a relatively low blood pressure of > 130 mmHg systolic or > 85 mmHg diastolic. In this patient category, there is no proof of better outcome with lowering blood pressure. However, the aim of our study was not to investigate the change in outcome of lowering blood pressure, but to study the pathophysiological reactions in patients with obesity and hypertension to different antihypertensive drugs. Moreover, not treating patients with a higher blood pressure and concomitant risk (metabolic syndrome) is unethical, and would undermine the careful design with a placebo period in this study.

Since many determinants were measured, some of the significant results might be false-positive due to multiple testing. However, determinants were not chosen and analyzed in a random way, but were chosen on their pathophysiological link with hypertension.

## Conclusions

The blood pressure-lowering response of antihypertensive medication is for aliskiren influenced by BMI and hsCRP, for HCTZ by heart rate and renin levels and for moxonidine by gender.

This emphasizes the multi-factorial mechanism of development of ORH, with activation of the RAAS system, sympathetic activation and volume overload being causative factors. Patient characteristics may guide choice of blood pressure-lowering medication in patients with ORH.

## Abbreviations

BMI: Body mass index
HCTZ: Hydrochlorothiazide
hsCRP: High-sensitivity C-reactive protein
MSNA: Muscle sympathetic nerve activity
ORH: Obesity-related hypertension
RAAS: Renin-angiotensin-aldosterone system
SAT: Subcutaneous adipose tissue
SBP: Systolic blood pressure
VAT: Visceral adipose tissue
